# Complex polyploid and hybrid species in an apomictic and sexual tropical forage grass group: genomic composition and evolution in *Urochloa* (*Brachiaria*) species

**DOI:** 10.1093/aob/mcab147

**Published:** 2021-12-07

**Authors:** Paulina Tomaszewska, Maria S Vorontsova, Stephen A Renvoize, Sarah Z Ficinski, Joseph Tohme, Trude Schwarzacher, Valheria Castiblanco, José J de Vega, Rowan A C Mitchell, J S (Pat) Heslop-Harrison

**Affiliations:** Department of Genetics and Genome Biology, University of Leicester, Leicester, UK; Royal Botanic Gardens, Kew, Richmond, Surrey, UK; Royal Botanic Gardens, Kew, Richmond, Surrey, UK; Royal Botanic Gardens, Kew, Richmond, Surrey, UK; International Center for Tropical Agriculture (CIAT), A.A. 6713, Cali, Colombia; Department of Genetics and Genome Biology, University of Leicester, Leicester, UK; Key Laboratory of Plant Resources Conservation and Sustainable Utilization/Guangdong Provincial Key Laboratory of Applied Botany, South China Botanical Garden, Chinese Academy of Sciences, Guangzhou, China; International Center for Tropical Agriculture (CIAT), A.A. 6713, Cali, Colombia; Earlham Institute, Norwich Research Park, Norwich, UK; Rothamsted Research, Harpenden, Hertfordshire, UK; Department of Genetics and Genome Biology, University of Leicester, Leicester, UK; Key Laboratory of Plant Resources Conservation and Sustainable Utilization/Guangdong Provincial Key Laboratory of Applied Botany, South China Botanical Garden, Chinese Academy of Sciences, Guangzhou, China

**Keywords:** Polyploidy, apomixis, repetitive DNA motifs, genome-specific sequences, evolution, *Brachiaria*, tropical forage grasses

## Abstract

**Background and Aims:**

Diploid and polyploid *Urochloa* (including *Brachiaria*, *Panicum* and *Megathyrsus* species) C_4_ tropical forage grasses originating from Africa are important for food security and the environment, often being planted in marginal lands worldwide. We aimed to characterize the nature of their genomes, the repetitive DNA and the genome composition of polyploids, leading to a model of the evolutionary pathways within the group including many apomictic species.

**Methods:**

Some 362 forage grass accessions from international germplasm collections were studied, and ploidy was determined using an optimized flow cytometry method. Whole-genome survey sequencing and molecular cytogenetic analysis were used to identify chromosomes and genomes in *Urochloa* accessions belonging to the ‘*brizantha*’ and ‘*humidicola*’ agamic complexes and *U. maxima*.

**Key Results:**

Genome structures are complex and variable, with multiple ploidies and genome compositions within the species, and no clear geographical patterns. Sequence analysis of nine diploid and polyploid accessions enabled identification of abundant genome-specific repetitive DNA motifs. *In situ* hybridization with a combination of repetitive DNA and genomic DNA probes identified evolutionary divergence and allowed us to discriminate the different genomes present in polyploids.

**Conclusions:**

We suggest a new coherent nomenclature for the genomes present. We develop a model of evolution at the whole-genome level in diploid and polyploid accessions showing processes of grass evolution. We support the retention of narrow species concepts for *Urochloa brizantha*, *U. decumbens* and *U. ruziziensis*, and do not consider diploids and polyploids of single species as cytotypes. The results and model will be valuable in making rational choices of parents for new hybrids, assist in use of the germplasm for breeding and selection of *Urochloa* with improved sustainability and agronomic potential, and assist in measuring and conserving biodiversity in grasslands.

## Introduction

Most arable crops have well-understood evolution and domestication processes, and the genetic diversity of their wild relatives is being exploited in breeding new varieties (Vaughan *et al.*, 2007). Native grasslands include high biodiversity that can be threatened by expansion of cultivated areas, while forage grasses occupy half the world’s agricultural land. Genomic knowledge is being increasingly applied to breeding the temperate *Lolium*–*Festuca* (ryegrass) complex (Velmurugan *et al.*, 2016), and there are a number of genetic selection and breeding programmes for tropical and sub-tropical forage (e.g. Worthington and Miles, 2015) but applications of omics-based technologies (Ishitani *et al.*, 2004) remain limited. The tropical forage grasses include clusters of species with various ploidies, and many reproduce through apomixis, but their genomic composition and diversity in general remain poorly characterized. The rational choice of parents for making crosses in breeding programmes, however, requires the knowledge of genome composition and ploidy. The integration of sequencing, molecular cytogenetic and bioinformatic tools allows the identification of genomes which come together in polyploids (Soltis *et al.*, 2013). Many crop species with polyploid members, including *Brassica* (Alix *et al.*, 2008) and the Brassicaceae (Cheng *et al.*, 2013), *Avena* (Tomaszewska and Kosina, 2018, 2021; Liu *et al.*, 2019) and particularly the tribe Triticeae (Hordeae) (Linde-Laursen *et al.*, 1997) have well-established genome designations (as single letters) to describe the ancestral genomes in auto- and allo-polyploids (amphiploids). Resolution of genome relationships in the wheat group has mainly assisted with extensive use of the germplasm pool in breeding (Feldman and Sears, 1981; Ali *et al.*, 2016; Rasheed *et al.*, 2018). Although it has proved difficult to identify conclusively the genomes present in *Urochloa* tropical forage grasses, some suggestions can be made based on a range of evidence (Corrêa *et al.*, 2020). Here, we aim to establish genome differences between diploids, and the genome composition in polyploids using advanced bioinformatic analysis of whole genome sequencing data to assist with genome nomenclature.

The pantropical grass genus *Urochloa* includes species previously classified under *Brachiaria*, *Megathyrsus*, and some *Eriochloa* and *Panicum* (Webster, 1987; González and Morton, 2005; Kellogg, 2015) and is a member of the Panicoideae tribe Paniceae, subtribe Melinidinae, comprising an estimated 150 annual and perennial grasses centred in sub-Saharan Africa (Kellogg, 2015; Soreng *et al.*, 2017). Joint missions in the early 1980s conducted by CGIAR (Consultative Group on International Agricultural Research) centres, CIAT (Centro Internacional de Agricultura Tropical) and ILRI (International Livestock Research Institute) in several African countries collected wild species mostly as live plant cuttings or ramets. These activities built a global grass collection with 700 accessions of *Urochloa* species representing a highly diverse gene pool for breeding and systematic studies (Keller-Grein *et al.*, 1996). Valuable traits of *Urochloa* include biomass yield, physiological tolerance to low-fertility acid soils of the tropics (Arroyave *et al.*, 2011), digestibility and energy content (Hanley *et al.*, 2020), insect tolerance (particularly to neotropical spittlebugs; Miles *et al.*, 2006) and disease resistance (Valério *et al.*, 1996; Alvarez *et al.*, 2014; Hernandez *et al.*, 2017). However, undesirable traits are also present, such as allelopathy (leaving bare soil; Kato-Noguchi *et al.*, 2014), cold-susceptibility (hybrid Mulato II: Pizarro *et al.*, 2013) and invasiveness (Durigan *et al.*, 2007 in the Brazilian Cerrado; *Urochloa panicoides* is on the US Federal Noxious Weed List https://www.aphis.usda.gov/plant_health/plant_pest_info/weeds/downloads/weedlist.pdf; accessed on 16 February 2021). These *Urochloa* grass collections have huge potential for sustainable improvement as well as conservation of grasslands, including pastures, rangelands, savannah, prairie, cerrado, and roadsides and verges, with various degrees of management of grazing. Breeding or trial programmes based in Colombia, Brazil, Thailand, Zimbabwe, Ethiopia, South Africa and Australia have led to the development of over a dozen cultivars (do Valle and Savidan, 1996; Singh *et al.*, 2010) and *Urochloa* is now the most widely planted forage grass in South America occupying 60 million hectares of grasslands in the tropical savannah ecoregion of Brazil (Gracindo *et al.*, 2014).

The extent of the monophyletic *Urochloa* lineage, encompassing most species previously placed in the genus *Brachiaria* on morphological grounds, is now established (Webster, 1987; Salariato *et al*., 2010, 2012). However, understanding of the genetic and genomic relationships within the diploid and polyploid species within the genus is limited. The species-level taxonomy within *Urochloa* established in African floras (Hutchinson and Dalziel, 1972; Clayton and Renvoize, 1982; Clayton, 1989) has not been fully maintained by recent floristic work (Sosef, 2016). Some *Urochloa* species have been arranged in agamic (apomictic) complexes: *U. brizantha*, *U. decumbens* and *U. ruziziensis* were classified into the ‘*brizantha*’ complex, and *U. humidicola* together with *U. dictyoneura* were assigned to the ‘*humidicola*’ complex (Lutts *et al.*, 1991; Renvoize and Maass, 1993). *Urochloa maxima* was previously assigned to *Megathyrsus* and *Panicum.* These species complexes have long been recognized as productive forages (Keller-Grein *et al.*, 1996). Some *Urochloa* species reproduce sexually, but others with apomictic or mixed reproduction allow odd levels of ploidy and contribute to increased intraspecific variability, making classification difficult. Some species are only known in the wild as diploids, but chromosome numbers of *U. ruziziensis* (Timbó *et al.*, 2014) and diploid *U. brizantha* (Pinheiro *et al.*, 2000) have been doubled in the laboratory to enable crossing with tetraploid apomictic species (Risso-Pascotto *et al.*, 2005; de Souza-Kaneshima *et al.*, 2010; Felismino *et al.*, 2010). The most common basic chromosome number is *x *= 9 (de Wet, 1986; Bernini and Marin-Morales, 2001), but *x *= 8, *x *= 7 (Basappa *et al.*, 1987) and *x *= 6 (Risso-Pascotto *et al.*, 2006; Boldrini *et al.*, 2009*b*; Worthington *et al.*, 2019) have been reported, making the genus *Urochloa* complex.

Characterization of the genome composition and diversity of *Urochloa* germplasm, phenotypes and ploidy is required for its effective use by researchers, breeders and farmers. Both whole genome sequencing and RNA-sequencing (RNAseq) (Higgins *et al.*, 2021) reveals unique repetitive and single-copy sequences present only in one genome and enables recognition and designation of diploid genomes and their relationships, and characterization of the genome composition in polyploids. Despite the agronomic importance of *Urochloa*, and the need to make crosses for breeding, genomes are not clearly defined (Boldrini *et al.*, 2009*a*), although some ploidy measurements have been made (Penteado *et al*., 2010; Jungmann *et al.*, 2010). The use of transposable element probes against *Urochloa* chromosomes indicates that many species are allopolyploid with differentiation in their transposable element composition (Santos *et al.*, 2015). Allopolyploidy is also shown by genetic analysis in apomicts (Worthington *et al.*, 2016) and genomic *in situ* hybridization (Corrêa *et al.*, 2020).

Here, we aimed to define the evolution and relationships of forage species in the tropical genus *Urochloa*, and understand evolutionary processes in polyploid, apomictic groups, and the diversification of abundant repetitive DNA sequences in their genomes. We measured ploidy in most of the *Urochloa* germplasm collection at CIAT (Colombia). We then aimed to use genomic and molecular cytogenetic approaches to identify repetitive DNA motifs and identify genome-specific sequences, to characterize the genomes present in the polyploid accessions (genomic composition), and to develop a model of evolution at the whole-genome level in diploid and polyploid accessions in the tropical forage grass group.

## MATERIALS AND METHODS

### Plant material

Studies were carried out on 362 accessions of *Urochloa* and related species (17 species, one synthetic hybrid, three unidentified accessions) focusing on material available on request for research and to breeders from CIAT and USDA (United States Department of Agriculture, USA) germplasm collections (Supplementary Data Table S1). Three notable unidentified accessions were included in our study: because of their unique characteristics they are already used in breeding programmes and the genomic composition must be analysed, along with our target species belonging to ‘*brizantha*’ and ‘*humidicola*’ agamic complexes and *U. maxima*, all having a huge potential for sustainable grazing and pasture management.

For diploids and polyploids, we use the narrow species concepts of Clayton and Renvoize (1982) and Clayton (1989) rather than the broader concepts of Sosef (2016) for ease of communication regarding the diverse genetic variants within *U. decumbens* and *U. ruziziensis*. Synonymy was updated and reconciled (POWO, 2019). For accessions from the CIAT and USDA germplasm collections, RNAseq data show that 111 lines (sampled from the 362 analysed here) are genetically distinct (Higgins *et al.*, 2021), supporting continued validity of correlation of collection locality with accession number, and a commendation to the CIAT germplasm resource collection group who maintained true lines through violent conflict, not allowing a small number of vigorous and robust lines to dominate the collection.

Fresh leaf material from apomictic and sexual plants was collected in the field in Colombia and trial plots grown at CIAT, and dried in silica gel according to the protocol presented by Tomaszewska *et al.* (2021). The leaf samples were then used to isolate nuclei for flow cytometry and extract whole genomic DNA. Seed samples for chromosome preparation and further cytogenetic studies were provided by CIAT (Colombia) and USDA (USA) (Table 1). Only one diploid species was used for chromosome preparation (*U. ruziziensis*). The reason for this was the lack of available diploid *U. brizantha* and *U. decumbens* seeds. Those diploid accessions supplied to us gave contradictory results, suggesting possible wrong assignment to species, and thus were excluded from further analysis. The use of polyploid species from the maintained collection for analysis of genomic composition yielded reliable results.

**Table 1.** List of accessions used in cytological studies, their chromosome numbers and ploidy levels

Species	Accession number	Donor	Number of chromosomes	Base number
*Urochloa* sp.	PI 657653^1^	USDA	2*n *= 4*x *= 32	8
*Urochloa* sp.	PI 508571^2^	USDA	2*n *= 4*x *= 36	9
*Urochloa* sp.	PI 508570^3^	USDA	2*n *= 5*x *= 45	9
*U. brizantha* (A.Rich.) R.D.Webster	PI 226049	USDA	2*n *= 6*x *= 54	9
*U. brizantha* (A.Rich.) R.D.Webster	PI 292187	USDA	2*n *= 4*x *= 36	9
*U. brizantha* (A.Rich.) R.D.Webster	PI 210724^4^	USDA	2*n *= 4*x *= 36	9
*U. decumbens* (Stapf) R.D.Webster	6370	CIAT	2*n *= 4*x *= 36	9
*U. decumbens* (Stapf) R.D.Webster	664	CIAT	2*n *= 4*x *= 36	9
*U. humidicola* (Rendle) Morrone & Zuloaga	16867	CIAT	2*n *= 8*x*+2/9*x*-4 = 50	6
*U. humidicola* (Rendle) Morrone & Zuloaga	26151	CIAT	2*n *= 6*x *= 36	6
*U. ruziziensis* (R.Germ. & C.M.Evrard) Crins	6419	CIAT	2*n *= 2*x *= 18	9
*U. maxima* (Jacq.) R.D.Webster	PI 284156	USDA	2*n *= 4*x *= 32	8
*U. maxima* (Jacq.) R.D.Webster	6171	CIAT	2*n *= 4*x *= 32	8
*U. maxima* (Jacq.) R.D.Webster	16004	CIAT	2*n *= 4*x *= 32	8

^1^ *Urochloa* sp. PI 657653 was received by USDA as *Panicum miliaceum*, re-identified at first as *Panicum sumatrense*, and finally described as *Urochloa* sp. We failed to determine the species identity of PI 657653, which has spikelets like *Urochloa adspersa* but leaves like *U. decumbens*, so does not match any species and may be of hybrid origin, and hence the difficulties in its taxonomic identification.

^2^ *Urochloa* sp. PI 508571 was not identified.

^3^ *Urochloa* sp. PI 508570 morphologically corresponds to *Urochloa panicoides*.

^4^Accession PI 210724 previously assigned to *Urochloa decumbens* has been re-identified as *U. brizantha.*

### Ploidy determination

Flow cytometry was conducted to establish ploidy levels of 362 accessions of *Urochloa* and related species (355 accessions from the CIAT germplasm collection, and seven accessions from the USDA germplasm collection; Supplementary Data Table S1). Cell nuclei from dehydrated leaf tissues were isolated mechanically, using the method described by Doležel *et al*. (2007) with some modifications following Tomaszewska *et al.* (2021). Approximately 500 mg of tissue was chopped with razor blade in a Petri dish containing 1 mL lysis buffer (0.1 m Tris, 2.5 mm MgCl_2_ x 6H_2_O, 85 mm NaCl, 0.1 % Triton X-100; pH = 7.0) supplemented with 15 mm β-mercaptoethanol and 1 % PVP-40 to reduce negative effects of cytosolic and phenolic compounds. The nuclear suspension was recovered and filtered through a 50-µm nylon mesh (CellTrics, Partec) to remove cell fragments and large debris, and then stained with 50 µg mL^−1^ propidium iodide (PI), supplemented with 50 µg mL^−1^ RNase to prevent staining of double-stranded RNA. Samples were incubated on ice and analysed within 10 min in an Accuri C6 Flow Cytometer (Becton Dickinson at the Flow Cytometry Facility, University of Leicester), equipped with a 20-mW laser illumination operating at 488 nm. The results were acquired using the CFlow Plus software. The software was set up according to Galbraith and Lambert (2012). The flow cytometry measurements were standardized following the methods described by Tomaszewska *et al.* (2021). Ploidy levels of *Urochloa* were estimated by comparing the relative fluorescence values of the peak positions of PI-stained nuclei (FL) of target samples to that of an external standard, following the protocol presented by Tomaszewska *et al.* (2021). The coefficient of variation (CV) of the G_0_/G_1_ peak was evaluated in each sample to estimate nuclei integrity and variation in DNA staining.

### DNA extraction and sequencing

Genomic DNA was extracted from fresh and dried leaves with the standard cetyltrimethylammonium bromide (CTAB)-based method (Doyle and Doyle, 1990). Whole genomic DNA from nine *Urochloa* accessions of various ploidies (Supplementary Data Table S2) was sequenced commercially (Novogene) with Illumina HiSeq 2× 150-bp paired-end reads (~12 Gb) (with a mean coverage of ~13×). Apomictic lines used by the breeders were selected for sequencing. Our aim was to generate universal probes that can be used on multiple accessions, not just sexual reproducing individuals that produce seeds (as used for chromosome preparations).

Project data have been deposited at the National Center for Biotechnology Information (NCBI; https://www.ncbi.nlm.nih.gov/sra/) under BioProject PRJNA771228.

### Identification and analysis of repetitive DNA sequences

Whole genome sequencing data were used to discover the most abundant repeats, and establish genomic compositions of *Urochloa* accessions of different ploidy levels. The whole genome shotgun sequence from *U. ruziziensis* cultivar CIAT 26162 (deposited in SRA under accession PRJNA437375; Worthington *et al.*, 2021) was used as a reference genome. Highly abundant repetitive DNA sequences were extracted as high-frequency 50-mers using the program Jellyfish v.2.2.6 (Marçais and Kingsford, 2011). Similarity-based clustering, repeat identification and classification of a subset of raw reads were performed using RepeatExplorer (RE; Novak *et al.*, 2013) and TAREAN (Novak *et al.*, 2017). All potential specific sequences extracted as 50-mer repeats or clusters were mapped to the reference genome (*U. ruziziensis,*Worthington *et al.*, 2021) and paired reads from nine sequenced genomes (Supplementary Data Table S2) using the program Geneious (Kearse *et al.*, 2012). The 50-mer repeats, contigs and clusters were analysed by BLAST searches against the NCBI database to check for repeat identification (Sayers *et al.*, 2019). The polymerase chain reaction (PCR) primer pairs were designed using Primer3 (Rozen and Skaletsky, 1999), and are listed in Supplementary Data Tables S3 and S4.

### Probes used for in situ hybridization

Four different types of probes were used for fluorescence *in situ* hybridization (FISH):

1. Two ribosomal DNA sequences: pTa71 (Gerlach and Bedbrook, 1979), which contains a 9-kb *Eco*RI fragment of *Triticum aestivum* L. consisting of the 18S–5.8S–25S rRNA genes and the transcribed and non-transcribed intergenic spacer regions; and pTa794 (Gerlach and Dyer, 1980), which contains part of the *T. aestivum* 5S rRNA gene and spacer sequences.
2. Whole genomic DNA extracted from six diploid species (Table 2).
3. Conserved regions found by k-mer and RepeatExplorer analysis, and amplified in a standard PCR using newly designed genome-specific primers synthesized commercially (Sigma-Aldrich; Supplementary Data Tables S3 and S4).
4. Newly designed synthetic 50-mer oligonucleotide probes (Supplementary Data Table S3) synthesized commercially (Sigma-Aldrich).

**Table 2.** List of genomic DNA and genome-specific probes used for *in situ* hybridization

Name of probe	Description
gDNA_Ubriz1	Genomic DNA extracted from *U. brizantha*, CIAT 16341 (2*n *= 2*x *= 18)
gDNA_Udec1	Genomic DNA extracted from *U. decumbens*, CIAT 26305 (2*n *= 2*x *= 18)
gDNA_Udec2	Genomic DNA extracted from *U. decumbens*, CIAT 6133a (2*n *= 2*x *= 18)
gDNA_Uruz1	Genomic DNA extracted from *U. ruziziensis*, CIAT 26348 (2*n *= 2*x *= 18)
gDNA_Umax1	Genomic DNA extracted from *U. maxima*, CIAT 16049 (2*n *= 2*x *= 16)
gDNA_Umax2	Genomic DNA extracted from *U. maxima*, CIAT 6898 (2*n *= 2*x *= 16)
Uruz-spec1	Sequence found in *U. ruziziensis*, CIAT 26162 (2*n *= 2*x *= 18) by k-mer analysis
Udec2x-spec1	Sequence found in *U. decumbens*, CIAT 26305 (2*n *= 2*x *= 18) by RepeatExplorer
Udec2x-spec3	Sequence found in *U. decumbens*, CIAT 26305 (2*n *= 2*x *= 18) by RepeatExplorer
Udec2x-spec6	Sequence found in *U. decumbens*, CIAT 26305 (2*n *= 2*x *= 18) by RepeatExplorer
Udec4x-spec3	Sequence found in *U. decumbens*, CIAT 664 (2*n *= 4*x *= 36) by k-mer analysis
Ubriz-spec2	Sequence found in *U. brizantha*, CIAT 26032 (2*n *= 4*x *= 36), 26745 (2*n *= 4*x *= 36), 16292 (2*n *= 4*x *= 36) and 16290 (2*n *= 5*x *= 45) by k-mer analysis
Ubriz-spec3	Sequence found in *U. brizantha*, CIAT 26032 (2*n *= 4*x *= 36), 26745 (2*n *= 4*x *= 36), 16292 (2*n *= 4*x *= 36) and 16290 (2*n *= 5*x *= 45) by k-mer analysis
Uhum-spec1	Sequence found in *U. humidicola*, CIAT 26155 (2*n *= 6*x *= 36) by k-mer analysis
Uhum-spec3	Sequence found in *U. humidicola*, CIAT 26155 (2*n *= 6*x *= 36) by k-mer analysis
Uhum-spec7	Sequence found in *U. humidicola*, CIAT 26155 (2*n *= 6*x *= 36) by k-mer analysis
Uhum-spec12	Sequence found in *U. humidicola*, CIAT 26155 (2*n *= 6*x *= 36) by RepeatExplorer analysis

More details on genome-specific probes are given in Supplementary Data Tables S3 and S9. Results of *in situ* hybridization with selected probes are shown in Figs 5 and 6, and in Table 4. A summary of the research findings is presented in Fig. 7.

Probes from groups 1–3 were labelled with digoxigenin-11-dUTP or biotin-11-dUTP (Roche) using a BioPrime Array CGH, and then purified using a BioPrime Purification Module (Invitrogen), according to the manufacturers’ instructions. Fluorescent nucleotides were incorporated during commercial synthesis for probes from group 4.

### Chromosome preparation

Chromosome preparation was carried out according to Schwarzacher and Heslop-Harrison (2000). The root-tips were collected from plants cultivated in a glasshouse, treated with α-bromonaphthalene at 4 °C for 6 h to accumulate metaphases, and fixed in 3 : 1 ethanol/acetic acid. Fixed root-tips were washed in enzyme buffer (10 mm citric acid/sodium citrate) for 15 min, digested in enzyme solution: 20 U mL^–1^ cellulase (Sigma C1184), 10 U mL^–1^ ‘Onozuka’ RS cellulase (RPI C32400), and 20 U mL^–1^ pectinase (Sigma P4716 from *Aspergillus niger*; solution in 40 % glycerol) in 10 mm enzyme buffer, and squashed in 60 % acetic acid. Cover-slips were removed after freezing with dry ice. Slides were air-dried and used for *in situ* hybridization within 3 months.

### In situ hybridization procedure

FISH was carried out using the method described by Schwarzacher and Heslop-Harrison (2000), with minor modifications as described below. The hybridization mixture consisted of 50 % deionized formamide, 10 % dextran sulphate, 1 % sodium dodecyl sulphate, 2× SSC, probe(s) (2 ng μL^−1^ each) and 200 ng μL^−1^ salmon sperm DNA. Additional use of genomic DNA extracted from diploid species, as a blocker, did not give different *in situ* hybridization results. The hybridization mixture and the chromosome slides were denatured together in a hybridization oven for 7 min at 75 °C. Hybridization was performed at 37 °C overnight (for amplified conserved regions and genomic DNA probes) or 2 days (for 50-mer oligonucleotide probes). Post-hybridization washes were carried out at 42 °C: in 2× SSC for 2 min, in 0.1× SSC (for 50-mer oligonucleotide probes and amplified conserved regions) or 20 % formamide in 0.1× SSC (for genomic DNA probes) for 6 min, and 2× SSC for 20 min. Hybridization sites were detected with streptavidin conjugated to Alexa 594 (Life Technologies-Molecular Probes) and antidigoxigenin conjugated to fluorescein isothiocyanate (FITC; Roche Diagnostics). Slides were then counterstained with DAPI. Mounted slides were examined with a Nikon Eclipse 80i epifluorescence microscope, and photographs were taken using a DS-QiMc monochromatic camera, and NIS-Elements v.2.34 software (Nikon) and assembled in Photoshop (Adobe) using only software functions affecting the whole image.

## RESULTS

### Taxonomic identification and ploidy measurement

We studied 362 accessions of *Urochloa* and related genera (summary in Table 3), and verified these taxonomically using live plants in CIAT, Cali, Colombia and reference herbarium specimens at the Royal Botanic Gardens, Kew, UK, linked where available with collection localities in Africa, morphological traits and cultivar status (data collected from CIAT GenBank, Genesys database and archived reports; Supplementary Data Table S1).

**Table 3.** Number of analysed accessions and their distribution in the various levels of ploidy

Species	2*n* = 2*x*	2*n* = 4*x*	2*n* = 5*x*	2*n* = 6*x*	2*n* = 7*x*	2*n* = 8*x + *2 or 2*n* = 9*x* − 4	2*n* = 9*x*
*Andropogon gayanus*	1						
*Panicum coloratum*	1						
*Paspalum dilatatum*		1					
*Pennisetum polystachion*				1			
*Pennisetum purpureum*		1					
*Urochloa arrecta*		1					
*Urochloa brizantha*	6	59	25	2			
*Urochloa decumbens*	18	26		1			
*Urochloa dictyoneura*					1		
*Urochloa dura*				1			
*Urochloa humidicola*				16	33	1	3
*Urochloa jubata*	1	1					
*Urochloa maxima*	25	104					
*Urochloa nigropedata*		1					
*Urochloa plantaginea*	1						
*Urochloa platynota*	1						
*Urochloa ruziziensis*	26						
*Urochloa* hybrid		1					
*Urochloa* sp. 1			1				
*Urochloa* sp. 2		1					
*Urochloa* sp. 3		1					

The ploidy of studied accessions was measured (Supplementary Data Fig. S1) using flow cytometry of fluorescently stained nuclei from dried leaf materials with an optimized method (Tomaszewska *et al.*, 2021) achieving a CV (coefficient of variation of the G_0_/G_1_ peak) of typically 2–5 %. Ploidy levels of 2*x*, 4*x*, 5*x* and 6*x* for the ‘*brizantha*’ agamic complex, 6*x*, 7*x* and 9*x* for the ‘*humidicola*’ agamic complex, and 2*x* and 4*x* for *U. maxima* were found (Table 3). Some accessions differed from published values (Supplementary Data Table S1). *Urochloa ruziziensis* was only found as a diploid (2*n *= 2*x *= 18), while other species, such as *U. humidicola*, were found only as polyploids.

**Fig. 1.**
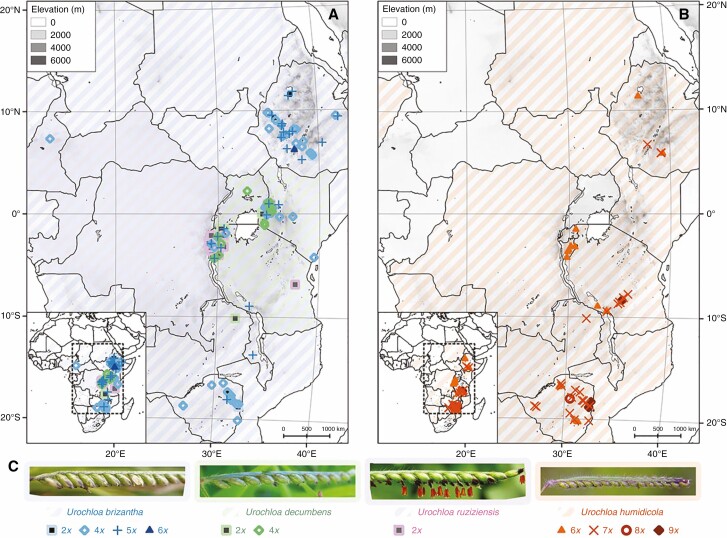
The natural distribution ranges of *Urochloa* (diagonal shading) and geographical origin of the accessions studied here (symbols). For relevant species, multiple ploidies were found at each collection location. (A) *Urochloa brizantha* is marked in blue, *U. decumbens* in green and *U. ruziziensis* in pink. (B) *Urochloa humidicola* is marked in orange. (C) Spikelet morphology. Diploid accessions are shown as squares, tetraploid as empty diamonds, pentaploid as upright crosses, hexaploid as triangles, heptaploid as diagonal crosses, octoploid as circles and nonaploid as filled diamonds. Natural distribution ranges are from wcsp.science.kew.org. Photograph of *U. humidicola* by Russell Cumming.

*Urochloa brizantha* and *U. maxima* are widespread in sub-Saharan Africa, the range of *U. decumbens* and *U. ruziziensis* is restricted to the area of Lake Victoria, and *U. humidicola* occurs from Nigeria eastwards to southern Ethiopia and southwards to South Africa. No correlation between the level of ploidy of the examined accessions of *U. brizantha*, *U. decumbens*, *U. ruziziensis* and *U. humidicola* with the area of their original East African collection sites was evident (Fig. 1). A mixture of species and ploidy levels was found at most collection sites; 4*x* and 5*x* accessions were predominant for *U. brizantha*, and 6*x* and 7*x* for *U. humidicola* (see also Table 3).

### Number of chromosomes and rDNA sites

**Fig. 2.**
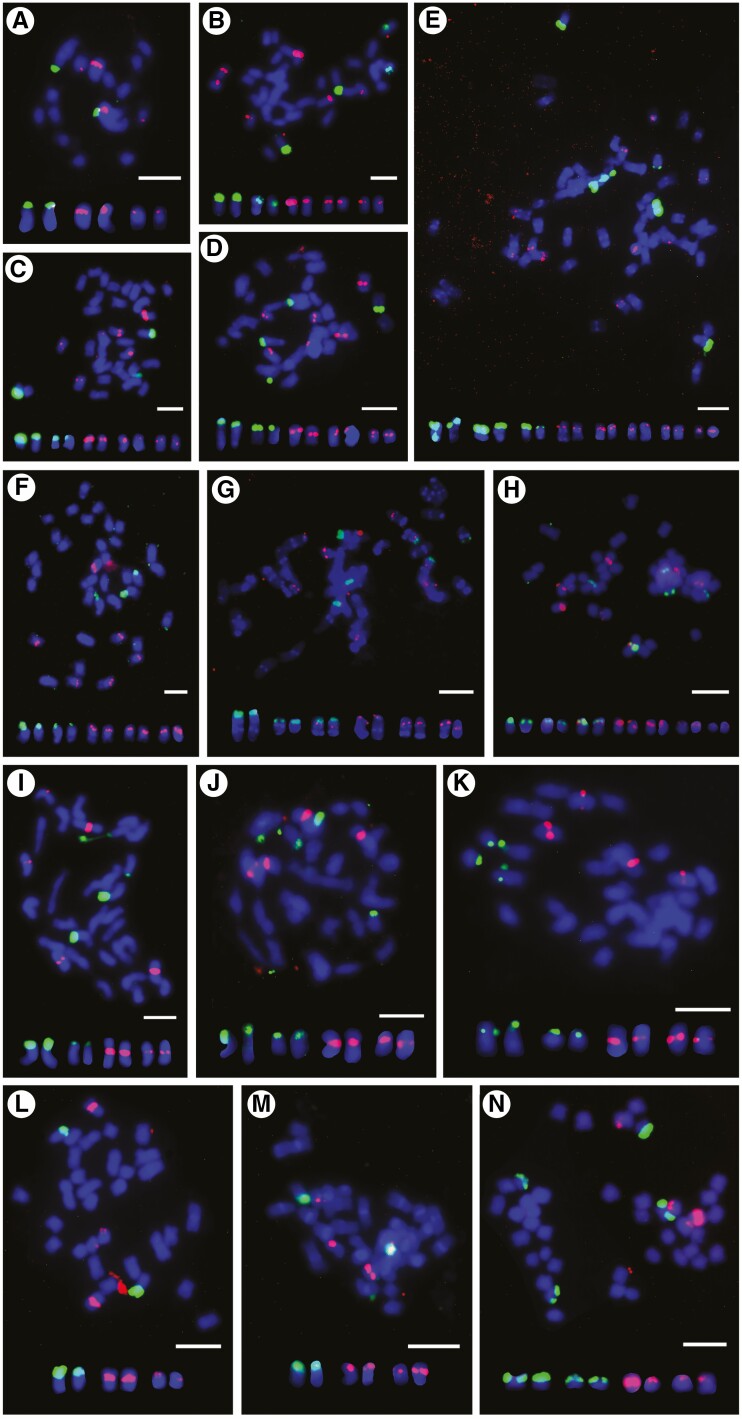
Localization of ribosomal 5S (red) and 18S–5.8S–25S (green) DNA sites on metaphase chromosomes of *Urochloa* species by fluorescence *in situ* hybridization. (A) *Urochloa ruziziensis* (2*x*; CIAT 6419); one pair of chromosomes with large 18S–5.8S–25S sites detected on the satellites, and two pairs of chromosomes with 5S sites observed in the interstitial regions. (B) *Urochloa decumbens* (4*x*; CIAT 664); 18S–5.8S–25S sites on satellites of two chromosome pairs, and 5S observed in the interstitial regions of three chromosome pairs; one pair of chromosomes showed much stronger 5S rDNA signals, as did diploid *U. ruziziensis*. (C) *Urochloa decumbens* (4*x*; CIAT 6370); same number and position of rDNA signals as in *U. decumbens* CIAT 664 (B). (D) *Urochloa brizantha* (4*x*; PI 210724); same number and position of rDNA signals as in tetraploid *U. decumbens* CIAT 664 (B) and CIAT 6370 (C). (E) *Urochloa brizantha* (6*x*; PI 226049); three pairs of chromosomes with 18S–5.8S–25S rDNA sites and five pairs of chromosomes with 5S rDNA sites. (F) *Urochloa brizantha* (4*x*; PI 292187); same number and position of rDNA signals as in tetraploid *U. decumbens* CIAT 664 (B) and CIAT 6370 (C). (G) *Urochloa humidicola* (6*x*; CIAT 26151); three pairs of chromosomes showed 18S–5.8S–25S rDNA signals on their satellites, and one pair was different, having additional 5S rDNA sites; another three pairs of chromosomes had 5S rDNA signals. (H) *Urochloa humidicola* (8*x *+ 2 or 9*x* − 4; CIAT 16867); three pairs of chromosomes showed 18S–5.8S–25S rDNA signals, and one pair had additional 5S rDNA sites; another four pairs of chromosomes had 5S rDNA signals. (I) *Urochloa maxima* (4*x*; CIAT 6171); two pairs of chromosomes with large 18S–5.8S–25S rDNA signals, and two pairs of chromosomes with 5S rDNA signals detected at the pericentromeric regions. (J) *Urochloa maxima* (4*x*; CIAT 16004); same number and position of rDNA signals as in *U. maxima* CIAT 6171 (I). (K) *Urochloa maxima* (4*x*; PI284156); same number and position of rDNA signals as in CIAT 6171 (I) and CIAT 16004 (J). (L) *Urochloa* sp. (4*x*; PI 657653); one pair of chromosomes with 18S–5.8S–25S rDNA signals and two pairs of chromosomes with 5S rDNA, which does not correspond to the pattern of rDNA signals in other tetraploids studied here. (M) *Urochloa* sp. (4*x*; PI 508571); same number of rDNA signals as in PI 657653 (L). (N) *Urochloa* sp. (5*x*; PI 508570); two pairs of chromosomes showing 18S–5.8S–25S rDNA and two pairs of chromosomes with 5S rDNA signals. Scale bars = 5µm.

The studied accessions were euploid with basic chromosome numbers of *x *= 6 for *U. humidicola*, *x *= 8 for *U. maxima*, and *x *= 9 for *U. brizantha*, *U. decumbens* and *U. ruziziensis* (Table 1) with the exception of one aneuploid accession of *U. humidicola* CIAT 16867 with 2*n *= 8*x *+ 2 or 9*x* − 4 = 50. Unidentified accessions had basic chromosome numbers of *x *= 8 and *x *= 9. FISH with a wheat 45S rDNA (18S–5.8S–25S; probe pTa71) and 5S rDNA (probe pTa794) (Fig. 2; details on number of rDNA sites in figure legend) showed, typically, one pair of major 45S rDNA sites per diploid chromosome complement, and two pairs of 5S rDNA sites on different chromosomes in species belonging to the ‘*brizantha*’ complex (Fig. 2A–E). Differences in the number and position of rDNA sites were not observed between studied accessions belonging to the ‘*brizantha*’ agamic complex. *Urochloa humidicola* had one pair of chromosomes showing both 45S and 5S rDNA signals and two pairs of chromosomes with only 45S rDNA signals (Fig. 2G, H). Two studied accessions of *U. humidicola* differed in number of 5S rDNA sites. In *U. maxima*, one pair of 45S rDNA sites and one pair of 5S rDNA sites per diploid chromosome complement were observed (Fig. 2I–K). The pattern of rDNA sites in unidentified accessions PI 657653 and PI 508570 did not correspond to the other polyploids studied here (Fig. 2L–N).

### Repetitive DNA motifs identified by k-mer and graph-based clustering of DNA sequence reads

The most abundant 50-mer repeats were extracted from 2 Gb of whole genome sequence reads from each of the ten accessions (our whole genome sequencing data from nine accessions listed in Supplementary Data Table S2 along with published whole genome shotgun sequence from *U. ruziziensis*, Worthington *et al.*, 2021). Those with sequence homology to rDNA, sequencing primers or chloroplast genomes, or with extreme GC ratio were omitted from further analysis as well as the telomeric repeat, (TTTAGGG)_n_, that represented about 0.1 % of the reads. The genome proportion of the remaining abundant 50-mers from reads or from assembled contigs of abundant 50-mers showed that most motifs were on the whole present with similar abundance in most *Urochloa* accessions (Supplementary Data Table S3; BLASTN search in Supplementary Data Table S5). Only a few motifs showed differences in abundance, indicating only limited species or genome-specific repeats are present. Comparison of abundant 50-mer motifs from the three diploid species, *U. ruziziensis* CIAT 26162, *U. decumbens* CIAT 26305 and *U. maxima* CIAT 16049, did not reveal any *U. decumbens*-specific repeats. However, some repeats showed different abundance: repeat 1392_24 was much more abundant in *U. ruziziensis* (1.18 %, Supplementary Data Table S3), and repeat 1101_42 represents 0.43 % of the diploid *U. maxima* genome (2*n *= 2*x *= 16), and has very low homology to *U. brizantha* and *U. humidicola* accessions. Repeat 1162_31 represents 1.72 % of the diploid *U. maxima* genome, and is also highly abundant in genomes of two out of five polyploid accessions of *U. brizantha*, representing slightly above 1 % of their genomes.

Since the whole genome sequence of diploid *U. brizantha* was not available, two k-mer strategies were used to find sequences potentially specific for the *U. brizantha* genome. In the first, the abundant 50-mer motifs from four polyploid accessions of *U. brizantha* were mapped to each other. Sequences that occurred in all four accessions were then *de novo* assembled. Contig 5 (Supplementary Data Fig. S2) with the highest genome proportion in *U. brizantha* accessions, but no or very low genome proportion in diploid *U. decumbens* and *U. ruziziensis*, was a candidate motif specific to the genome of *U. brizantha*. In the second strategy, we tested our hypothesis that tetraploid *U. decumbens* is an allopolyploid with the genomic composition XXYZ (where X, Y and Z represent genomes to be determined). We have not ruled out such a possibility because synthetic multi-generation hybrids involving *U. ruziziensis*, *U. decumbens* and *U. brizantha* are known (Supplementary Data Table S1), and such crosses could take place in nature. We mapped abundant unassembled 50-mer motifs from tetraploid *U. decumbens* CIAT 664, to 50-mer datasets from diploid *U. decumbens*, *U. ruziziensis* and *U. maxima*. The differentially abundant sequence 1771_76 (>100× the genome proportion in tetraploid *U. decumbens* and four polyploid accessions of *U. brizantha* compared to the diploids where it represented <0.01 % of the genome; Supplementary Data Table S3) was a candidate repeat specific to genome Z.

The 50-mer sequence dataset from *U. humidicola* (6*x*) was mapped to highly abundant 50-mers from the three diploid species and identified four motifs unique to *U. humidicola*: 5899, 1014, 1015_2 and 7000 (Supplementary Data Table S3). Similarly, to find abundant repetitive motifs in 4*x Urochloa* sp. (PI 657653, unidentified accession, potentially of hybrid origin), two 50-mer sequences were extracted, 1134_5 and 1644_4, representing 0.33 and 0.2 % of the genome, respectively, with abundance below 0.01 % in the diploids (*U. decumbens*, *U. maxima*, and already published *U. ruziziensis*, Worthington *et al.*, 2021).

For graph-based sequence clustering and characterization of repeats, 2-Gb subsets of raw sequence from each of the nine *Urochloa* accessions were analysed using RepeatExplorer and TAREAN (Novak *et al.*, 2013, 2017). Generally, 38.2–60.0 % of reads were assigned into clusters of related sequence reads (Supplementary Data Fig. S3; Supplementary Data Tables S6-S9). As with k-mers, sequences showing high homology to rDNA or chloroplast genomes, and extreme GC ratio were omitted from further analysis, and the final list of putative genome-specific sequences was created by comparing genome abundance between accessions (Supplementary Data Table S4). The number of raw reads with high homology to the most abundant clusters/RE motifs in each one of the sequence datasets were then counted for ten whole genome sequence reads. Those clusters showing a high proportion in one diploid genome are candidate genome-specific sequences (Supplementary Data Table S4), and some were selected for testing as probes by chromosomal *in situ* hybridization (see below and Table 2).

**Fig. 3.**
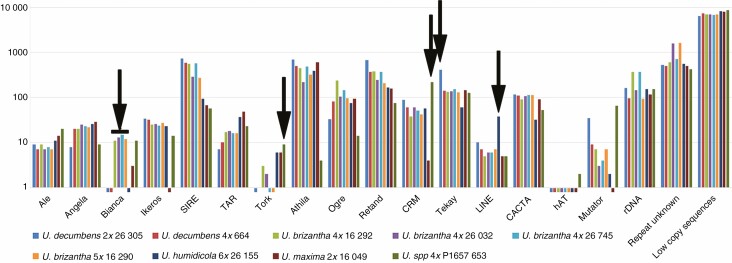
Relative abundance (log) of DNA sequence classes in *Urochloa* species and hybrids from whole-genome sequence reads. Automated repeat identification of graph-based clustering (RepeatExplorer, TAREAN) and abundant k-mer motifs were classified by nucleotide domain hits and database BLASTN searches (Supplementary Data Tables S7 and S9). Arrows indicate some motifs with differential abundance between accessions. Bars below 1 (*y*-axis) indicate abundance <0.01 % of genome.

Transposable elements were recognized in each of the nine sequenced genomes (Fig. 3; Supplementary Data Table S10; for *U. ruziziensis* see Worthington *et al.*, 2021). The automated annotation provided by RepeatExplorer will omit, or give incorrect, identification of some elements based on homology to known sequences; therefore, regardless of annotation, any elements differing in abundance between accessions were candidates for use as probes for *in situ* hybridization to distinguish genomes. Thus, sequences with differential abundance identified were the Bianca retrotransposon in *U. brizantha* polyploids, the highly abundant Tekay retrotransposon in diploid *U. decumbens*, the retrotransposon CRM in 4× PI 657653, and the long interspersed elements (LINE) in *U. humidicola* (arrows in Fig. 3). The Tork retrotransposon was found in some *U. brizantha*, suggesting differences in genome structure between accessions.

### Chromosomal organization and genome specificity of repetitive DNA sequences

**Fig. 4.**
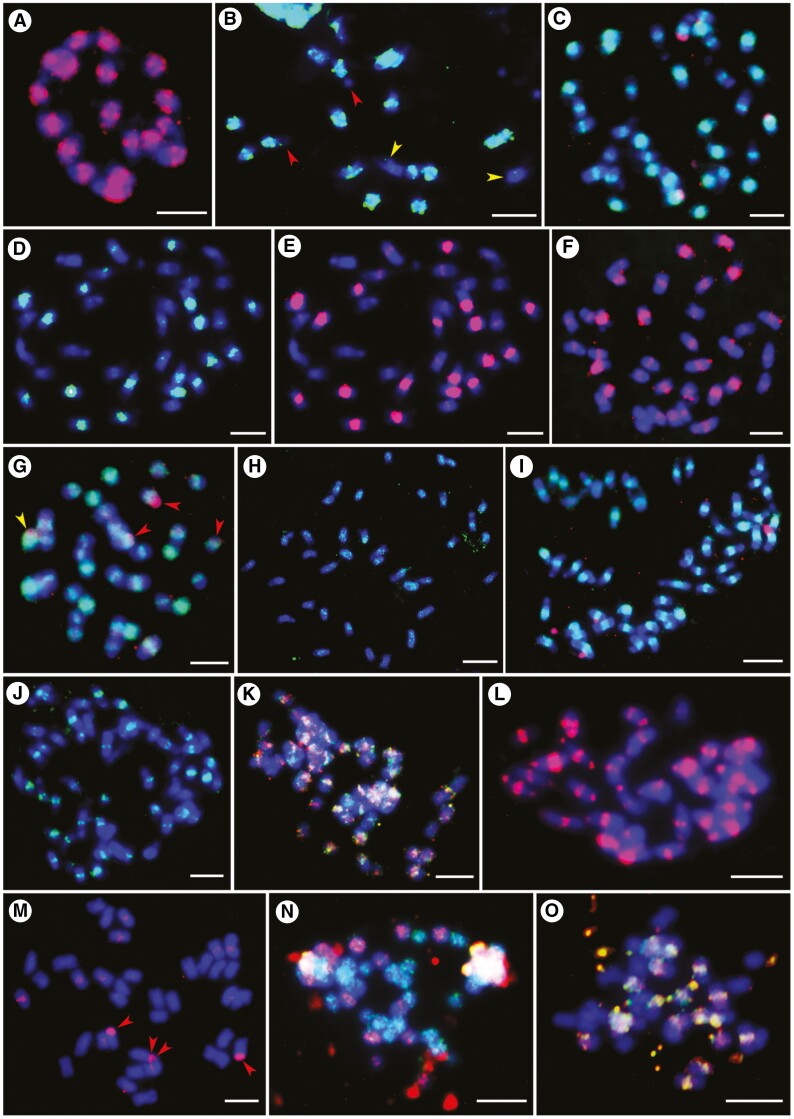
Localization of labelled whole genomic DNA (gDNA) from diploid species used as probes for *in situ* hybridization on metaphase chromosomes (fluorescing blue). (A) Eighteen chromosomes of *U. ruziziensis* (2*x*, CIAT 6419) showing strong signals of gDNA_Uruz1 probe (red) in pericentromeric regions of chromosomes and some terminal signals on one or both arms of several chromosomes. (B) *Urochloa ruziziensis* (2*x*, CIAT 6419) metaphase showing strong signals of gDNA_Udec1 probe (green) in eight pairs of chromosomes (satellites of two chromosomes remain unlabelled; red arrowheads), and weak signals in centromeres of two chromosomes (yellow arrowheads). (C) Metaphase of *U. decumbens* (4*x*, CIAT 6370) with 18 strong and 18 weak signals of gDNA_Udec2 probe (green), and four red signals of rDNA after hybridization with gDNA_Umax1 probe. (D) Metaphase of *U. decumbens* (4*x*, CIAT 6370) showing 18 strong and 18 weak signals of gDNA_Udec1 probe (green). (E) Same metaphase as in D, showing signals of gDNA_Uruz1 probe (red) in the same position as gDNA_Udec1 probe, which confirms the similarity of the genomes of these accessions. (F) Chromosomes of *U. brizantha* (4*x*, PI 292187) with red signals of gDNA_Uruz1 probe: nine very strong and 27 weak. (G) Chromosomes of *U. brizantha* (4*x*, PI 292187) with green signals of gDNA_Udec1 probe: nine very strong and 27 weak; gDNA_Umax1 probe shows four red signals (rDNA): one signal in chromosome with strong gDNA_Udec1 signal (yellow arrowheads) and three signals in chromosomes with weak gDNA_Udec1 signals (red arrowheads). (H) Metaphase of *U. brizantha* (4*x*, PI 292187) with some rDNA and dispersed signals of gDNA_Umax2 probe (green); gDNA_Ubriz1 probe did not show signals. (I) Metaphase of *U. brizantha* (6*x*, PI 226049) with gDNA_Uruz1 probe signals (green) in centromeres: 18 strong, 18 weaker and 18 weak; gDNA_Ubriz1 probe (red) gave four red signals (rDNA). (J) Metaphase of *U. brizantha* (6*x*, PI 226049) with gDNA_Udec1 probe signals (green) in centromeres: 18 strong, 18 weaker and 18 weak. (K) Metaphase of *U. humidicola* (8*x *+ 2 or 9*x* − 4, CIAT 16867) showing dispersed signals of gDNA_Uruz1 (red) and gDNA_Udec2 (green) probes along chromosomes; many of these signals did not overlap. (L) Metaphase of *U. maxima* (4*x*, CIAT 6171) with pericentromeric and telomeric signals of gDNA_Umax1 probe (red). (M) Chromosomes of *U. decumbens* (4*x*, CIAT 664) with four rDNA (red arrowheads) and very weak dispersed signals in centromeres after hybridization with gDNA_Umax1 probe (red). (N) Metaphase of *Urochloa* sp. (5*x*, PI 508570) showing 18 chromosomes with dispersed signals of gDNA_Ubriz1 probe (red) and 27 chromosomes with dispersed signals of gDNA_Umax1 probe (green). (O) Metaphase of *Urochloa* sp. (4*x*, PI 508571) showing signals of gDNA_Uruz1 probe (red) and gDNA_Udec1 probe (green) in 18 chromosomes; the 18 remaining chromosomes showed no signals. Scale bars = 5 µm.

**Fig. 5.**
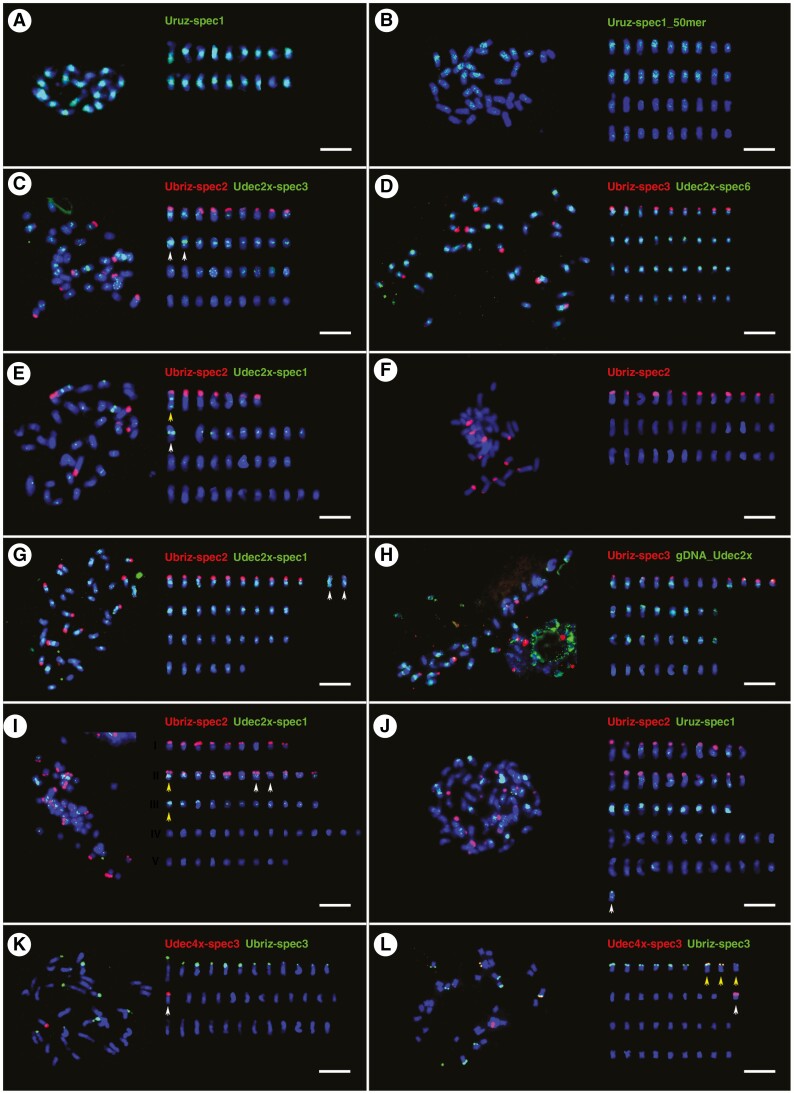
Localization of abundant repetitive sequences on chromosomes of different species belonging to the ‘*brizantha*’ complex. Probes are described in Supplementary Data Table S3. Chromosomes (right) were arranged by chromosomal distribution of FISH signals and chromosome length. (A) Eighteen strong signals of Uruz-spec1 probe (green) at pericentromeric regions of *U. ruziziensis* (2*x*, CIAT 6419) chromosomes. (B) Uruz-spec1_50mer labelled 18 chromosomes of *U. decumbens* (4*x*, CIAT 664; green). (C) Ubriz-spec2 probe showed strong signals in terminal regions of nine chromosomes of *U. decumbens* (4*x*, CIAT 664). Udec2x-spec3 probe showed strong signals in pericentromeric positions of chromosomes, even those with terminal Ubriz-spec2 signals. Two chromosomes exhibited very strong green fluorescence (white arrows). Some signals were more dispersed along chromosomes, and the 18 chromosomes without Ubriz-spec2 signal had weak Udec2x-spec3 signals. (D) Metaphase of *U. decumbens* (4*x*, CIAT 664); Ubriz-spec3 showed a similar pattern of signals as Ubriz-spec2 in C. Udec2x-spec6 showed only nine chromosomes with weak signals. (E) Metaphase of *U. decumbens* (4*x*, CIAT 6370); Ubriz-spec2 probe produced seven signals in the terminal position of chromosomes; one chromosome with (yellow arrow) and one chromosome without Ubriz-spec2 signals (white arrow) showed strong signals in pericentromeric and subtelomeric positions of Udec2x-spec1 probe. (F) Metaphase of *U. brizantha* (4*x*, PI 210724); 12 signals of Ubriz-spec2 probe at terminal regions of chromosomes. (G) Metaphase of *U. brizantha* (4*x*, PI 292187); same number and position of Ubriz-spec2 signals as in F where two of the 12 signals were weaker (white arrows). Thirty chromosomes showed strong to weak Udec2x-spec1 signals, while the other six had very weak or no signals. (H) *Urochloa brizantha* (4*x*, PI 292187); gDNA-Udec probe gave strong signals on some chromosomes with Ubriz-spec3 signals and those without Ubriz-spec3 signals. (I) *Urochloa brizantha* (6*x*, PI 226049); Ubriz-spec2 and Udec2x-spec1 probes differentiated chromosomes into five types: nine chromosomes with Ubriz-spec2 signals (group I), 11 chromosomes with Ubriz-spec2 and Udec2x-spec1 signals (group II), 11 chromosomes with strong Udec2x-spec1 signals (group III), 14 chromosomes with very weak Udec2x-spec1 signals (group IV), and nine chromosomes without any signals (group V). In group II, there was a pair of chromosomes showing the same pattern of signals (white arrows), although it seems that another chromosome from this group (yellow arrow) had the same strong pericentromeric signal of Udec2x-spec1 probe as another chromosome from group III (yellow arrow). (J) Metaphase of *U. brizantha* (6*x*, PI 226049) showing nine chromosomes with very strong pericentromeric signals of Uruz-spec1 probe. (K) Metaphase of *U. brizantha* (4*x*, PI 292187); Ubriz-spec3 probe gave 12 signals at the terminal position of chromosomes. One very strong signal of Udec4x-spec3 detected at the terminal region of one chromosome (white arrow). (L) Metaphase of *U. decumbens* (4*x*, CIAT 664) showing four terminal signals of Udec4x-spec3 probe: one strong on chromosome without Ubriz-spec3 signals, and three weak on chromosomes showing Ubriz-spec3 signals (white arrows). Scale bars = 5 µm.

Total genomic DNAs (gDNA; Table 2) from diploid species of *U. brizantha* (Ubriz), *U. decumbens* (Udec), *U. ruziziensis* (Uruz) and *U. maxima* (Umax) were used as probes for genomic *in situ* hybridization (GISH) on 14 accessions of *Urochloa* diploids and polyploids (Table 1). The results are given in Table 4, and example micrographs are shown in Fig. 4 (giving details regarding probe combinations and observed signals in the legend). In summary, probes gDNA_Uruz and gDNA_Udec showed signals in broadly pericentromeric regions rather than painting whole chromosomes, and the differential hybridization conditions (hybridization stringency 72 and 85 %; using only salmon sperm DNA or together with gDNA extracted from diploid species as an additional block of cross-hybridization of common sequence motifs) did not affect GISH results. Different strengths of signals in pericentromeric position of chromosomes in polyploids belonging the to ‘*brizantha*’ complex indicated that these species might be allopolyploids (Fig. 4C–J). Further investigation using genome-specific probes showed that polyploids from the ‘*brizantha*’ agamic complex are allopolyploids, but the signal strengths of gDNA probes were not sufficient to recognize genomes (see the last paragraph of this section, and the legend to Fig. 5). The simultaneous use of probes gDNA_Uruz and gDNA_Udec against chromosomes of *U. humidicola* showed differential dispersed signals on many chromosomes, indicating the differences between diploid *U. ruziziensis* and *U. decumbens* genomes (Fig. 4K). The gDNA_Ubriz probe hybridized to rDNA sites of different species, but not to pericentromeric regions of chromosomes (Fig. 4I; Table 4). The gDNA_Umax probe showed very strong pericentromeric signals on all 32 chromosomes of tetraploid *U*. maxima, in addition to terminal and subterminal regions (Fig. 4L). *Urochloa* accessions not assigned to species are clearly allopolyploids (Fig. 4N, O; Table 4). Ultimately, our GISH results were difficult to interpret, and thus there was a need to develop specific probes to gain the much-needed genome specificity.

**Table 4.** Description of genomic *in situ* hybridization results

Accession	Genomic DNA probes			
	gDNA_Ubriz	gDNA_Udec	gDNA_Uruz	gDNA_Umax
*U. ruziziensis* CIAT 6419	No signal	Signals in pericentromeric position of 18 chromosomes; same position of signals as gDNA_Uruz probe	Signals in pericentromeric position of 18 chromosomes; same position of signals as gDNA_Udec probe	No signal
*U. decumbens* PI 210724	Four 45S rDNA signals	Strong signals in pericentromeric position of 18 chromosomes, and weak signals in pericentromeric position of remaining 18 chromosomes; same position of signals as gDNA_Uruz probe	Strong signals in pericentromeric position of 18 chromosomes, and weak signals in pericentromeric position of remaining 18 chromosomes; same position of signals as gDNA_Udec probe	Four 45S rDNA signals
*U. decumbens* CIAT 664	Four 45S rDNA signals	Strong signals in pericentromeric position of 18 chromosomes, and weak signals in pericentromeric position of remaining 18 chromosomes; same position of signals as gDNA_Uruz probe	Strong signals in pericentromeric position of 18 chromosomes, and weak signals in pericentromeric position of remaining 18 chromosomes; same position of signals as gDNA_Udec probe	Four 45S rDNA signals
*U. decumbens* CIAT 6370	Four 45S rDNA signals	Strong signals in pericentromeric position of 18 chromosomes, and weak signals in pericentromeric position of remaining 18 chromosomes; same position of signals as gDNA_Uruz probe	Strong signals in pericentromeric position of 18 chromosomes, and weak signals in pericentromeric position of remaining 18 chromosomes; same position of signals as gDNA_Udec probe	Four 45S rDNA signals
*U. brizantha* PI 292187	Four 45S rDNA signals	Nine chromosomes painted; 27 chromosomes show pericentromeric signals; same position of signals as gDNA_Uruz probe	Nine chromosomes painted; 27 chromosomes show pericentromeric signals; same position of signals as gDNA_Udec probe	Four 45S rDNA signals
*U. brizantha* PI 226049	Six 45S rDNA signals	Eighteen strong, 18 weaker and 18 weak signals in pericentromeric position of chromosomes	Eighteen strong, 18 weaker and 18 weak signals in pericentromeric position of chromosomes	Six 45S rDNA signals
*U. humidicola* CIAT 26151	Six 45S rDNA signals	Interspersed signals along chromosomes	Interspersed signals along chromosomes	Six 45S rDNA signals
*U. humidicola* CIAT 16867	Six 45S rDNA signals	Interspersed signals along chromosomes	Interspersed signals along chromosomes	Six 45S rDNA signals
*U. maxima* CIAT 6171	Four 45S rDNA signals	Four 45S rDNA signals	Four 45S rDNA signals	Thirty-two chromosomes show signals in telomeric–subtelomeric and pericentromeric position
*U. maxima* CIAT 16004	Four 45S rDNA signals	Four 45S rDNA signals	Four 45S rDNA signals	Thirty-two chromosomes show signals in telomeric–subtelomeric and pericentromeric position
*U. maxima* PI 284156	Four 45S rDNA signals	Four 45S rDNA signals	Four 45S rDNA signals	Thirty-two chromosomes show signals in telomeric-subtelomeric and pericentromeric position
*Urochloa* sp. PI 657653	Four 45S rDNA signals, weak signals in centromeres	Four 45S rDNA signals	Four 45S rDNA signals	Signals along 16 chromosomes
*Urochloa* sp. PI 508571	No signal	Signals in pericentromeric position of 18 chromosomes; same position of signals as gDNA_Uruz probe	Signals in pericentromeric position of 18 chromosomes; same position of signals as gDNA_Udec probe	No signal
*Urochloa* sp. PI 508570	Signals along 18 chromosomes; some chromosomes show gDNA_Ubriz and gDNA_Umax signals	Four 45S rDNA signals	No signal	Signals along 27 chromosomes

**Fig. 6.**
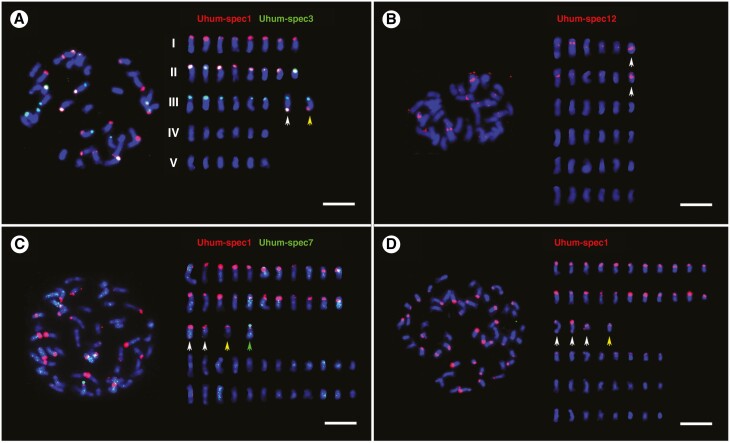
Localization of abundant repetitive sequences on chromosomes of *U. humidicola* accessions. Probes are described in Supplementary Data Table S3; Chromosomes (right) were arranged by chromosomal distribution of FISH signals and chromosome length. (A) Uhum-spec1 and Uhum-spec3 probes differentiated chromosomes of *U. humidicola* (6*x*, CIAT 26151) into four types: eight chromosomes with terminal Uhum-spec1 signals (group I), eight chromosomes with Uhum-spec1 and Uhum-spec3 signals (group II), eight chromosomes with Uhum-spec3 signals (group III), and 12 chromosomes without any signals (group IV). Two chromosomes belonging to group III differed from the other six: one of them had two additional signals of Uhum-spec1 and Uhum-spec3 probes (white arrow), while the other had only one additional signal of Uhum-spec1 probe (yellow arrow). (B) *Urochloa humidicola* (6*x*, CIAT 26151) showed signals of Uhum-spec12 probe at pericentromeric and intercalary position of 12 chromosomes. The intensity and distribution of these signals indicated the presence of six pairs of chromosomes. In particular, one pair of shorter chromosomes exhibited very strong pericentromeric signals of Uhum-spec12 (white arrows). (C) *Urochloa humidicola* (8*x *+ 2 or 9*x* − 4, CIAT 16867); Uhum-spec7 signals were dispersed along chromosomes, some of which were more intensive, but it is difficult to deduce if there was any specific pattern of their distribution (high stringency conditions). Uhum-spec1 probe showed signals on 26 chromosomes, but four chromosomes were different, showing additional signals: two chromosomes had extra Uhum-spec1 signals on the opposite arms (white arrows), one chromosome showed doubled Uhum-spec1 signal (yellow arrow), and one chromosome had strong terminal Uhum-spec7 signal (green arrow). (D) Chromosomes of *U. humidicola* (8*x *+ 2 or 9*x* − 4, CIAT 16867); the low stringency conditions, allowing hybridization between DNAs sharing 72 % sequence identity, revealed eight additional weak signals of Uhum-spec1 probe. Three chromosomes had Uhum-spec1 signals on both arms (white arrows), and one chromosome showed signals on one arm (yellow arrow). Scale bars = 5 µm.

**Fig. 7.**
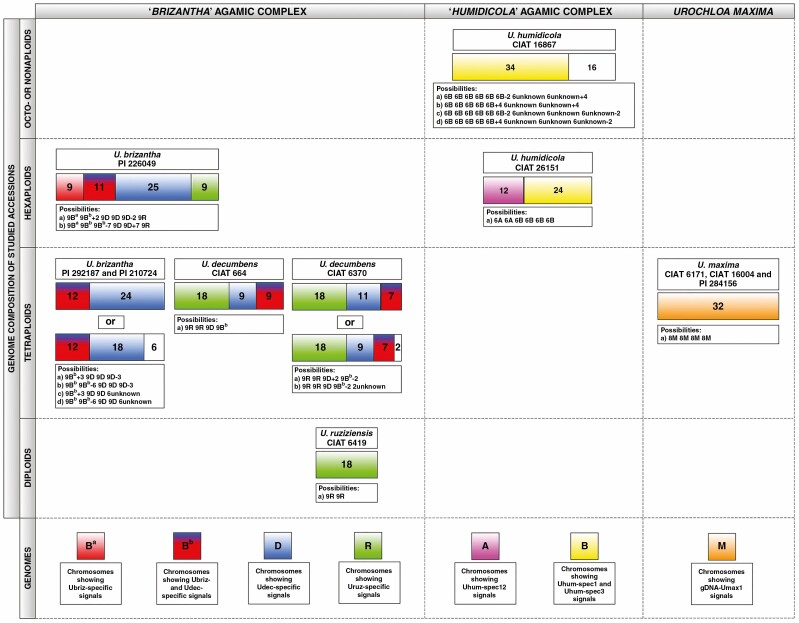
Summary of *in situ* hybridization results with gDNA and genome-specific probes. Inferred genomic composition (coloured blocks) and chromosome numbers (shown) of studied accessions belonging to the ‘*brizantha*’ (left) and ‘*humidicola*’ (middle) complexes, and *U. maxima* (right). White blocks with numbers: undetermined or diverged genome. Probe names are given in Table 2. Details of the probes are given in Supplementary Data Tables S3 and S9.

Probes designed from highly abundant sequences recognized by k-mer (Supplementary Data Table S3), and RepeatExplorer and TAREAN (Supplementary Data Table S4) analyses were used, mostly in differential pairs, for *in situ* hybridization to localize repeats on *Urochloa* chromosomes, and distinguish genomes in polyploids (see Fig. 5 for *‘brizantha*’ and Fig. 6 for ‘*humidicola*’ complexes; signal summary in Supplementary Data Tables S3 and S4: chromosomes were grouped by signal location and intensity). Overall, *in situ* hybridization strength correlated with *in silico* analysis (percentage of sequence in the genomes), now showing the genome and chromosomal distribution of the probes and enabling discrimination of the genome of origin of most chromosomes in the polyploid accessions. The Uruz-specific probe perfectly labelled 18 chromosomes belonging to the diploid *U. ruziziensis*, allowing us to also recognize this genome in polyploids. All putative Ubriz-specific probes designed using two strategies gave the same number and position of signals; and some chromosomes shared both Ubriz- and Udec-specific signals. Uhum-specific probes enabled recognition of all genomes which come together in hexaploid *U. humidicola*. Detailed descriptions of the probes and hybridization results are given in the extended legend (Figs 5 and 6) and probe description in Supplementary Data Tables S3 and S4. A summary of the *in situ* hybridization results is presented in Fig. 7, showing the possible genome composition of the studied accessions.

## Discussion

Through our analysis of repetitive DNA sequences using unassembled raw-reads, molecular cytogenetic and flow cytometry tools, we were able to define the nature and similarity between the *Urochloa* species and genomes available internationally in germplasm resource collections. By identifying repetitive sequences that were unique to the different genomes present in the species, and identifying distinct genomes in the polyploids, we revealed the genome composition of polyploids and the nature of evolutionary changes in the primary DNA sequence of repetitive motifs and changes in their abundance. Together with growth habit and morphological data, evaluation of the *Urochloa* material confirmed the challenges in defining the genetic relationships of the accessions used in forage breeding. Analyses of data including collection localities in Africa, morphological traits and cultivar status, together with ploidy levels and sequencing data are critical for understanding biodiversity in the wild, and using diverse genebank material in breeding.

### Ploidy and geographical origin

All the species analysed here are native to sub-Saharan Africa (Renvoize and Maass, 1993). Information regarding the collection sites, most from the international 1984/85 expeditions representing the majority of germplasm in Colombia and Brazil, and reintroductions within Africa (Wassie *et al.*, 2018), allowed us to correlate geographical distribution and ploidy levels as determined by flow cytometry. For *Urochloa* species with multiple ploidies, representatives of all ploidies were found in each geographical region, indicating co-occurrence, no major niche specialization, and the opportunity for hybridization and introgression, including segmental allopolyploidy. This is not uncommon for species with multiple ploidies. In wild *Tripleurospermum inodorum* (Asteraceae) in central Europe, for example, Čertner *et al*. (2017) studying the spatio-temporal patterns of ploidy coexistence found tetraploid cytotypes alone in about half or more of the populations, diploids in about 10 % of populations, with the remaining populations being a mixture of ploidies. Natural selection may produce polyploids and hybrids with strong geographical signals (Hagl *et al.*, 2021; Alix *et al.*, 2017). Even in species with no significant ecological differences between cytotypes (e.g. in *Aster amellus*), no mixing of ploidies is seen even in contact zones (Mandáková and Münzbergová, 2006). Deliberate or accidental roadside or forage introductions (likely to be over-represented in the genebank material sampled here) may introduce different ploidies, although our accessions are genetically different (Hanley *et al.*, 2020; Higgins *et al.*, 2021). Polyploids are often argued to have a competitive advantage over diploids (Alix *et al.*, 2017) and production of polyploid seeds and individuals by diploids is widespread, although subsequent establishment of whole polyploid populations and their expansion can be hindered by insufficient seed production (Levin, 2021). Thus, it is not surprising that multiple ploidy levels (2*x*–9*x*) in many collection areas, including new polyploids and fertile 3*x* hybrids, were found and suggests co-existence of the various ploidy levels in both *U. brizantha* and *U. humidicola*.

### Chromosome and genome differentiation in Urochloa polyploids

The karyotypes of three *Urochloa* species belonging to the ‘*brizantha*’ complex show little differences, having chromosomes similar in size and morphology (Bernini and Marin-Morales, 2001; Nielen *et al.*, 2010). Physical mapping of 5S and 18S–5.8S–25S rDNA locations provides a chromosome marker, but the mostly similar patterns in the ‘*brizantha*’ complex did not assist in identification of genome composition (see Fig. 2; Akiyama *et al.*, 2010; Nielen *et al.*, 2010; Santos *et al.*, 2015; Nani *et al.*, 2018). In the three accessions with desirable agronomic characteristics that could not be assigned to species based on morphology, the number of rDNA sites did not correspond to ploidy, with only one pair of 45S rDNA sites in the two tetraploids (two pairs of sites expected), and four 45S sites in a pentaploid (expectation five), suggesting a more complex origin involving processes such as karyotype reorganization, aneuploidy or segmental allopolyploidy and introgression.

Using two diploid total genomic, gDNA, probes (Uruz and Udec) to chromosomes of three species belonging to the ‘*brizantha*’ complex, *in situ* hybridization results showed very small differences in hybridization patterns between groups of chromosomes (candidate genomes), with strong signals in centromeres consistent with Corrêa *et al.* (2020). However, the genome-specific motifs identified in sequence data (see below) suggested that some chromosomes sharing similar pericentromeric signals actually belong to different genomes. Polyploid *U. humidicola* showed dispersed signals of gDNA probes along all chromosomes, making it impossible to discriminate genomes. GISH indicated that tetraploid *U. maxima* is autopolyploid, which is in contrast to the other polyploids in *Urochloa* that have been identified as allopolyploids. The autopolyploid origin of *U. maxima* and its facultative apomixis type of reproduction have been proved by different authors (Toledo-Silva *et al.*, 2013; Lara *et al.*, 2019), meaning that gDNA probes here showing both terminal and pericentromeric signals are informative.

Santos *et al.* (2015) revealed some differentiation of candidate genome-specific Ty3-gypsy retrotransposons in pericentromeric regions of *Urochloa* chromosomes. *Urochloa* contrasts with another Poaceae, *Avena*, where GISH can characterize individual genomes (Katsiotis *et al.*, 2000; Tomaszewska and Kosina, 2021), ‘painting’ most of the chromosomal lengths. The *Urochloa* results indicate that bulk repetitive sequences present in the gDNA probes have diverged only slightly in sequence and copy number during speciation of the diploid *U. brizantha* ancestors combined in polyploids, showing only weak genome specificity (Corrêa *et al.*, 2020). Centromeres of plants are often composed of abundant tandemly repeated sequences and sometimes centromere-specific retrotransposon families (e.g. the CR family in grasses; Miller *et al.*, 1998; Presting *et al.*, 1998; Heslop-Harrison and Schwarzacher, 2011). The centromere-specific distribution pattern of signals of genomic (this study; Corrêa *et al.*, 2020) and transposable element (Santos *et al.*, 2015) probes in *Urochloa* may be due to the retrotransposons being clustered in centromeres and thus generating strong signals, whereas copies located along chromosome arms are dispersed (Miller *et al.*, 1998).

While GISH did not differentiate *Urochloa* genomes, bioinformatic analysis of unassembled raw DNA sequences identified short sequence motifs that showed differential abundance among accessions. *In situ* hybridization of the various motifs to metaphase chromosomes confirmed the differential abundance and enabled identification of the genomes present in polyploids, leading to a model of *Urochloa* evolution (see below). All the sequences were present on multiple chromosomes, showing both amplification and dispersion or homogenization of the motifs after speciation from a common ancestral *Urochloa* genome, and each sequence had a characteristic proximal, distal or more dispersed chromosomal location. However, in contrast to a parallel analysis in *Avena* species (Liu *et al.*, 2019), no major DNA satellite or tandem repeats giving chromosomal bands were revealed in *Urochloa*. Triticeae species with much larger genomes and chromosomes have many tandem repeats, including simple sequence motifs, that are tribe-, genus- or species-specific and have been widely used to identify chromosomes (along with total genomic DNA; e.g. Ali *et al.*, 2016; Patokar *et al.*, 2016). More generally, in a wide range of species, repetitive sequences have been identified as a key component of evolutionary mechanisms and karyotypic differentiation, playing an important role in speciation (Heslop-Harrison and Schwarzacher, 2011; Mehrotra and Goyal, 2014). Comparison of GISH, and the sequences and chromosomal distribution of repetitive sequences identified by cloning or sequence analysis, suggests considerable differences in repetitive sequence evolution between taxonomic ‘groups’ (family, tribe or genus). It is evident that each group has distinctive rules for chromosome and repetitive sequence evolution, but these are not easily transferrable as models between species groups.

### Taxonomy and the genomic composition of Urochloa polyploids

**Fig. 8.**
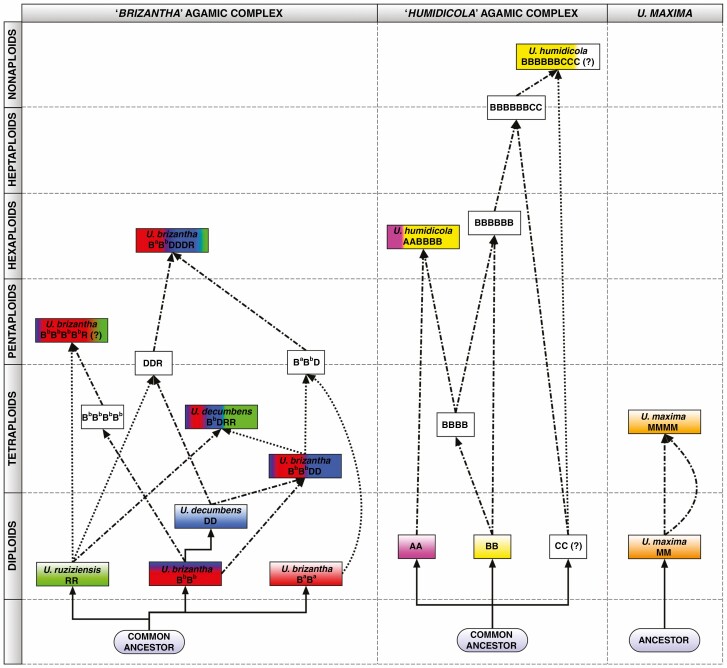
A model for the evolutionary origin of *Urochloa* species in the ‘*brizantha*’ (left) and ‘*humidicola*’ (middle) complexes, and in *U. maxima* (right), built from this study and published data: genome sizes and ploidy (Supplementary Data Table S1); repetitive DNA sequences from whole genome sequence analysis (k-mer counts and graph-based clustering; Supplementary Data Tables S3 and S4); *in situ* hybridization with defined repeat probes (Figs 5 and 6) and genomic DNA (Fig. 4; and Corrêa *et al.*, 2020); karyotype analyses (Corrêa *et al.*, 2020); meiotic behaviour (Risso-Pascotto *et al.*, 2005; Mendes-Bonato *et al.*, 2007; Fuzinatto *et al.*, 2007); chloroplast genome (Pessoa-Filho *et al.*, 2017); hybrid occurrence (Table 1; Mendes *et al.*, 2006; Vigna *et al.*, 2016; Risso-Pascotto *et al.*, 2005); CIAT breeding programmes (Renvoize and Maass, 1993; Miles *et al.*, 1996); and reported apomixis (Roche *et al.*, 2001). The three line types show evolutionary sequence divergence (solid line), and hybridization events involving haploid, *n* (dotted line), or unreduced, 2*n* (dash-dotted line), gametes from different genomes (designated in Fig. 7). White blocks: putative species/hybrids.

Species concepts for many of the genebank accessions of *Urochloa* (including *Brachiaria*, and other species which have previously been placed in the genera *Megathyrsus*, *Eriochloa* and *Panicum*) have been problematic, not least because of the range of ploidies, apomixis, vegetative propagated lines, intermediate morphological traits and growth habits, and the presence of hybrids occurring in the wild or as landraces selected by forage grass breeders and farmers. Our results support the maintenance of distinct species for *U. ruziziensis*, *U. brizantha*, *U. decumbens*, *U. humidicola* and *U. maxima* (chromosomal organization in Figs 4–6; relationship models in Figs 7 and 8). We accept the species concepts for diploids (Clayton and Renvoize, 1982; Clayton, 1989), and do not consider allopolyploids as cytotypes.

Following the genome labelling system adopted across the Triticeae (Hordeae) tribe (Linde-Laursen *et al.*, 1997) or in *Brassica* (Cheng *et al.*, 2013; Alix *et al.*, 2008), the level of genomic differentiation as found here by extensive sequence and chromosomal analysis is high enough that we propose designating basic genomes in *Urochloa* using the upper-case letters R, B and D for the ‘*brizantha*’ complex, rather than the superscript designations B^b^, B^d^ and B^r^ (Corrêa *et al.*, 2020) for *U. brizantha*, *U. decumbens* and *U. ruziziensis*, which would indicate a much closer relationship of the three genomes than we think is present. Similarly, we suggest use of A and B or even C (for ‘*humidicola*’ complex), and M for *U. maxima* (Figs 7 and 8). More limited differentiation allows us to suggest use of superscript designations, referring to modified basic genomes, for less-well differentiated genomes including B^a^ and B^b^. Figure 7 illustrates the chromosome and genome composition of the accessions studied here. *Urochloa ruziziensis* was diploid; *U. brizantha* with multiple polyploid levels shows a variation of chromosomes and genomes, as does *U. decumbens*. An important question to be answered is whether allopolyploid species should be considered separate species or not, since their genomic composition indicates that they are of hybrid origin and their parental species are known?

Our analysis supported the genome composition of hexaploid *U. humidicola* (based on meiotic behaviour, Vigna *et al.*, 2016; and transposable elements, Santos *et al.*, 2015) as including A and B genomes (and probably the C genome in higher ploidy levels). Ty1-gypsy Tat probe (Santos *et al.*, 2015) and Uhum-spec12 (Fig. 6B) are good markers for the A genome. The B genome is more variable in showing three types of chromosomes.

### Evolutionary model for Urochloa species

Three substantive models (Fig. 8) to explain the evolution of *Urochloa* polyploids in the ‘*brizantha*’ and ‘*humidicola’* agamic complexes, and *U. maxima* were generated from multiple lines of evidence. Renvoize and Maass (1993) suggested that diploid *U. decumbens* evolved from *U. brizantha*: the natural range of *U. decumbens* covers the area of a candidate ancestral *U. brizantha* form or variety (e.g. *U. brizantha* var. *latifolium* Oliver or *U. brizantha* var. *angustifolia* Stent & Rattray) with lanceolate hairy leaves and a decumbent habit. We found genome-specific repetitive sequences in *U. decumbens*, but all of them were shared with *U. brizantha*, supporting the order of evolutionary branching. These data contradict Basappa *et al*.’s (1987) suggestion that *U. decumbens* is a natural hybrid between *U. brizantha* and *U. ruziziensis*, and confirmation of this hypothesis would be meiotic abnormalities found in *U. decumbens*. We support this hypothesis for tetraploid *U. decumbens*, but not the diploid accession we studied. Our results were inconclusive for the hexaploid *U. brizantha* accessions (see Fig. 7). Pessoa-Filho *et al.* (2017) found that tetraploid *U. brizantha* and *U. decumbens* show high similarity of their plastid sequences and low number of single nucleotide polymorphisms, which may suggest that a single polyploidization event took place to establish both the tetraploid *U. brizantha* and *U. decumbens*: namely a potential fertilization of a tetraploid *U. brizantha* BD gamete and an unreduced RR gamete of a diploid *U. ruziziensis.*

Risso-Pascotto *et al.* (2006) suggested that hexaploid *U. brizantha* probably resulted from ‘chromosome doubling of a triploid derived from species that did not display the same behaviour for spindle organization’. Triploid hybrids were found in nature (Timbó *et al.*, 2014), and may originate from crosses between diploid *U. ruziziensis* and tetraploid *U. decumbens* or *U. brizantha*. Thus, a hexaploid species would be created by crossing two different triploids rather than doubling of genomes of a triploid hybrid. This suggestion arises from the presence of only one R genome in the hexaploid *U. brizantha*, as indicated by our *in situ* hybridization analysis (see Figs 4, 5 and 7). We also suggest, based on our *in situ* hybridization and repetitive sequence composition in hexaploid *U. brizantha*, that there are at least two cytotypes/varieties of diploid *U. brizantha*. Another possibility is that the genomes of the hexaploid *U. brizantha* have undergone structural changes after polyploidization, and therefore some chromosomes show signals of both *U. brizantha*- and *U decumbens*-specific probes, and some only show *U. brizantha*-specific signals. This hypothesis can be supported by Bernini and Marin-Morales (2001) and Nielen *et al.* (2010), who showed differences in karyotypes of diploid and tetraploid *U. brizantha* accessions.

The most likely evolution of species belonging to the ‘*humidicola*’ complex is much more difficult to propose, because all accessions are polyploid and there is no suggestion as to which diploid species may be considered ancestral. There are three known levels of ploidy in this species: hexaploid, heptaploid and nonaploid (Boldrini *et al.*, 2009*a*; Jungmann *et al.*, 2010; Vigna *et al.*, 2016; we also had an inconclusive accession 2*n *= 8*x *+ 2 or 9*x* − 4 = 50). Our analysis of the genomic composition of the hexaploid species matches with meiotic analyses conducted by Boldrini *et al.* (2009*b*) and Vigna *et al.* (2016), and the model of evolution of species belonging to the ‘*humidicola*’ complex is supported by *in situ* hybridization with genome-specific probes (see Fig. 6). The B genome includes chromosomes showing three different types of signals, which may suggest that *U. humidicola* has gone through several rounds of polyploidization. Broader analysis of genome composition of species belonging to the ‘*humidicola*’ agamic complex, including different accessions of *U. humidicola* and *U. dictyoneura*, would be desirable to understand the process of speciation, especially as tetraploid accessions with 2*n *= 4*x *= 24 are known (Boldrini *et al.*, 2010) and could have contributed to the evolution of *U. humidicola*, which shows odd ploidy levels.

Our *in situ* hybridization studies gave evidence for potential introgression within *Urochloa.* Some polyploid lines (*U. brizantha* and *U. humidicola*) here have chromosome pairs that are different from others within their genome (see Figs 5G, J–L and 6A, C, D), resembling segmental allopolyploidy (Mendes-Bonato *et al.*, 2002) or disomic introgression lines. Frequent introgression seems to occur in wheat (Cheng *et al.*, 2019) and oat polyploids, and in breeding, whole chromosomes, chromosome arms or segments may be substituted. An example is *Triticale,* which may have not the expected seven chromosome pairs of each genome but 14 A, 12 B, two D and 14 R chromosomes (Neves *et al.*, 1997). Some hybrid species are diploid or reduce chromosome numbers so they are not clearly tetraploid – *Petunia hybrida* is 2*n *= 14, like its ancestors (Bombarely *et al.*, 2016), with a mixture of ancestral genomes, while the octaploid *Nicotiana* cell fusion hybrid (4*x* + 4*x*) has lost a few chromosomes (Patel *et al.*, 2011).

## Conclusions

Genome composition and evolution are complex in *Urochola* tropical forage grasses. Grasslands are not only a major source of food production but also provide environmental services: water, soil preservation, carbon capture, etc., often in more biodiverse regions, where identification of species and their relationships will assist in grass conservation. Despite their lower economic value, breeding and exploitation of biodiversity is required within the group (whether using sequence data or a genetic map, for example as in *Lolium*, Tomaszewski *et al.*, 2012). Like wheat and *Brassica* crops, wild relatives contribute to the current pool of diversity used in *Urochloa* tropical forage grass improvement, with additional complexities from apomixis. Knowledge of genome relationships and polyploid genome composition gives opportunities for rational and systematic use of accessions in forage improvement programmes (superdomestication: Vaughan *et al.*, 2007). Complementing our study showing the diversification of genomes and repetitive DNA, a parallel study (Hanley *et al.*, 2020) found high levels of genetic diversity in 20 genes related to forage quality in 104 of the accessions studied here.

Our study was focused on accessions available from international germplasm collections to breeders and researchers. As Keller-Grein *et al.* (1996) correctly pointed out, further collecting of the *Urochloa* species in Africa would be worthwhile to enrich the germplasm collection with new accessions, finding further useful characteristics that can be exploited, and to better understand its complicated evolution, adding to the analysis here. For legal regulations regarding biosecurity restrictions (diseases and invasive species) and Plant Breeders Rights and germplasm ownership, it is necessary to have an accepted name for every species, and our identification of genomes and genome composition in *Urochloa* polyploids presents the necessary framework.

Breeding programmes often work with a single ploidy because directed crosses among parents with different ploidies are challenging. We suggest that *Urochloa* species are all part of a common gene pool, and any hybrid combination might be possible and become a successful forage variety, noxious weed or disease host. The current breeding programmes at CIAT manage tetraploid interspecific crosses within the ‘*brizantha*’ agamic complex, hexaploid crosses within the ‘*humidicola*’ agamic complex and tetraploid intraspecific crosses of *U. maxima*. The choice of appropriate strategies to generate hybrids requires knowledge of ploidy provided by our research, supported by the model of evolution and diversification of the species.

## SUPPLEMENTARY DATA

Supplementary data are available online at https://academic.oup.com/aob and consist of the following. Fig. S1. Ploidy measured by flow cytometry of PI-stained nuclei from dehydrated tissues of diploid, tetraploid, pentaploid and hexaploid accessions of *Urochloa* showing very sharp peaks. Fig. S2. Contig 5 as a candidate motif specific to the *U. brizantha* genome. Fig. S3. Distribution of graph-based clusters. Table S1. List of accessions used in the study, their ploidy levels, growth habits and geographical distribution. Table S2. Summary of sequencing data quality. Table S3. Potential genome-specific 50-mer sequences, their genome proportion, and description of probes and *in situ* hybridization signals. Table S4. Potential genome-specific repeats and their genome proportion. Table S5. BLASTN search of highly abundant potential genome-specific 50-mers. Table S6. NCBI BLASTN results of clusters found using RepeatExplorer. Table S7. RepeatExplorer characterization of selected repeat clusters of *Urochloa* accessions. Table S8. NCBI BLASTN results of clusters found using TAREAN. Table S9. TAREAN characterization of selected repeat clusters of *Urochloa* accessions. Table S10. Repetitive DNA composition of *Urochloa* genomes.

mcab147_suppl_Supplementary_Table_S1

mcab147_suppl_Supplementary_Table_S2

mcab147_suppl_Supplementary_Table_S3

mcab147_suppl_Supplementary_Table_S4

mcab147_suppl_Supplementary_Table_S5

mcab147_suppl_Supplementary_Table_S6

mcab147_suppl_Supplementary_Table_S7

mcab147_suppl_Supplementary_Table_S8

mcab147_suppl_Supplementary_Table_S9

mcab147_suppl_Supplementary_Table_S10

mcab147_suppl_Supplementary_Figure_S1

mcab147_suppl_Supplementary_Figure_S2

mcab147_suppl_Supplementary_Figure_S3
