# Supplementary figures and images for: Complex polyploid and hybrid species in an apomictic and sexual tropical forage grass group: genomic composition and evolution in Urochloa (Brachiaria) species
Source: Ann Bot. 2021 Dec 7;131(1):87–108. doi: 10.1093/aob/mcab147 (PMC9904353; doi:10.1093/aob/mcab147)

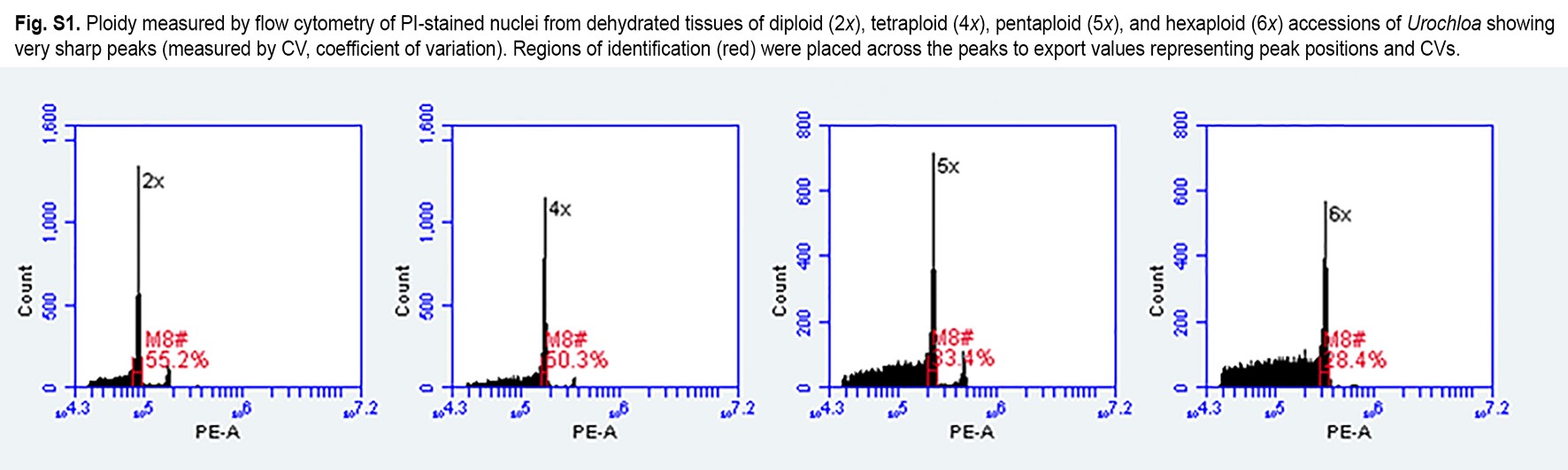

Supplement: mcab147_suppl_Supplementary_Figure_S1 [file mcab147_suppl_supplementary_figure_s1.jpeg]

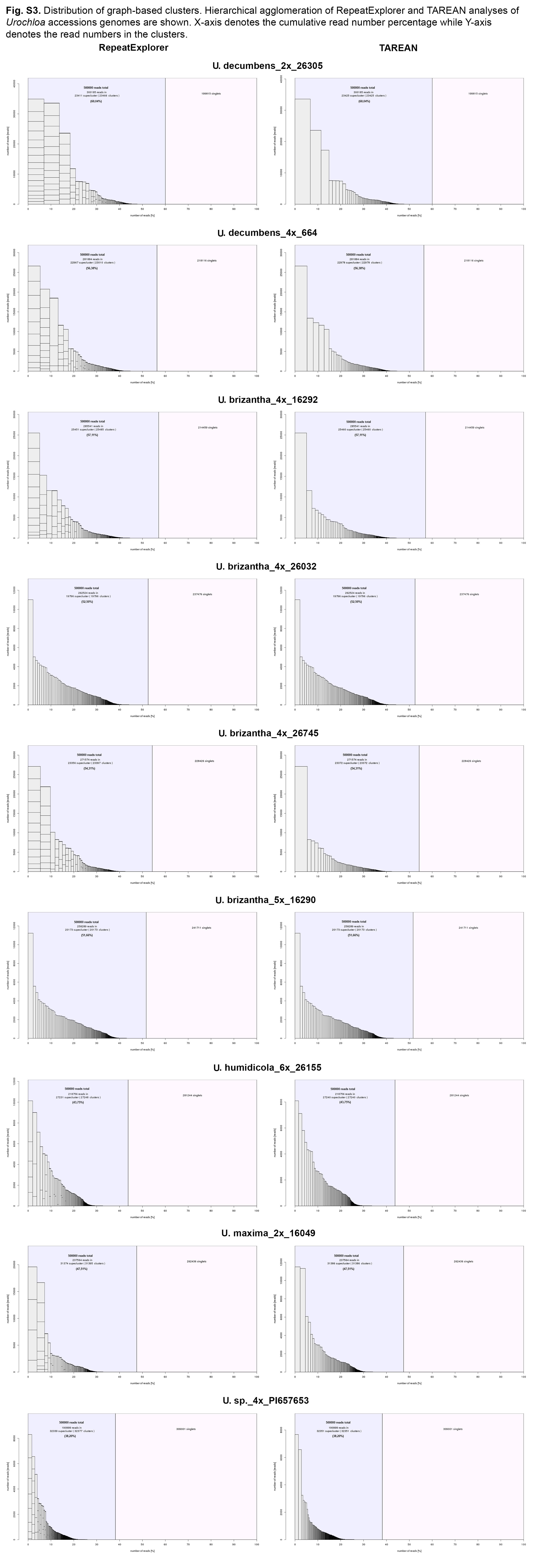

Supplement: mcab147_suppl_Supplementary_Figure_S3 [file mcab147_suppl_supplementary_figure_s3.jpeg]
