# Supplementary material for: Complex polyploid and hybrid species in an apomictic and sexual tropical forage grass group: genomic composition and evolution in Urochloa (Brachiaria) species
Source: Ann Bot. 2021 Dec 7;131(1):87–108. doi: 10.1093/aob/mcab147 (PMC9904353; doi:10.1093/aob/mcab147)
Supplement: mcab147_suppl_Supplementary_Figure_S2 [file mcab147_suppl_supplementary_figure_s2.docx]

**Fig. S2. Contig 5 as a candidate motif specific to *U. brizantha* genome**

**Contig5_U.brizantha** AGAATGTTGTCTGCTACACAGGGCGAGGCAAATTTACATGAATAGAGCTATCATGATTTTTTAGAAAAAAA=TAATAACTCCCACATGTCCATAGAGGTACCCCTTGGGCATGCAGCGTCGCAGCTCACGAGAGGGGCTCCAGCGCACTACCATGGATGGTCCACATCTCAAGTAGTAGTCGAGGAACACCAAGACATGGTATGTTAACTCATATGTTAATTTCGGGTTGTGGACCAAACGTCTAGTCCATAGTCTTGGCGTCCGAGTGGGTTTCGACGGTCAAAATTGATTAACTTCCCCATGAATTTCCATAACTTATTCGTTTGGAGCCCGAATCAATCGCGTTTTTT

Length: 350 bp & 1 gap

GC: 44.6%

**Genome proportion:**

|  | *U. brizantha*  CIAT 26745 (4*x*) | *U. brizantha*  CIAT 26032 (4*x*) | *U. brizantha*  CIAT 16292 (4*x*) | *U. brizantha*  CIAT 26745 (5*x*) |
| --- | --- | --- | --- | --- |
| Contig 5 | 1,03% | 0,26% | 0,33% | 0,92% |
